# Ultra-processed foods consumption is associated with multiple sclerosis severity

**DOI:** 10.3389/fneur.2023.1086720

**Published:** 2023-01-24

**Authors:** Monica Guglielmetti, Giuseppe Grosso, Cinzia Ferraris, Roberto Bergamaschi, Eleonora Tavazzi, Alessandro La Malfa, H. Al-Qahtani Wahidah, Anna Tagliabue

**Affiliations:** ^1^Department of Public Health, Experimental and Forensic Medicine, Human Nutrition and Eating Disorder Research Center, University of Pavia, Pavia, Italy; ^2^Laboratory of Food Education and Sport Nutrition, Department of Public Health, Experimental and Forensic Medicine, University of Pavia, Pavia, Italy; ^3^Department of Biomedical and Biotechnological Sciences, University of Catania, Catania, Italy; ^4^Center for Human Nutrition and Mediterranean Foods (NUTREA), University of Catania, Catania, Italy; ^5^Neurological Institute-Foundation IRCCS Casimiro Mondino, Pavia, Italy; ^6^Department of Food Sciences and Nutrition, College of Food and Agriculture Sciences, King Saud University, Riyadh, Saudi Arabia

**Keywords:** multiple sclerosis, ultra-processed food, multiple sclerosis severity, dietary habits, disability, NOVA classification

## Abstract

**Background:**

MS is a chronic inflammatory neurological and immune-mediated disease of multifactorial etiology. Ultra-processed foods (UPFs) have been generally considered unhealthy due to their poor nutritional value. Emerging evidence suggests that factors other than their nutritional content may play an additional role toward chronic inflammation.

**Aim:**

To investigate the potential association of UPF consumption and MS severity in a group of MS Italian consecutive patients.

**Methods:**

Demographic (age, sex, marital status, educational level), neurological (EDSS, MSSS), and nutritional (anthropometric measures, dietary habits) information were collected. Physical activity and smoking habits were also investigated. Food items were grouped according to the NOVA classification. Patients were classified in two groups based on MS severity (“mild” and “moderate to high”).

**Results:**

Higher UPF consumption was associated with moderate-to-high MS severity compared to lower consumption in both the unadjusted model (OR = 2.28, 95% CI: 1.04–5.01) and after adjustment for potential background (OR = 2.46, 95% CI: 1.04–5.83) and clinical confounding factors (OR = 2.97, 95% CI: 1.13–7.77).

**Conclusions:**

Although these results are only preliminary and hypothesis generating, it is important to explore how various aspects of the diet may relate to MS severity in order to identify the best strategy to support MS patients over the disease course.

## 1. Introduction

Multiple sclerosis (MS) is a chronic inflammatory neurological and immune-mediated disease of multifactorial etiology. MS is one of the most important causes of disability in young adults (1) and it affects more women than men (with a female-male incidence ratio ranging between 1.5:1 and 2.5:1). The specific mechanisms through which MS is caused are still unknown but it is clear that it derives from a combination of genetic and environmental factors (2–4). Several studies reported the possible role of diet as a risk factor for MS and its progression (5–7). The possible role of dietary components on neuroinflammation, one of the main pathogenetic mechanisms in MS, has gained a lot of interest, especially since researchers have focused their interest on intestinal microbiota and the gut-brain axis. Diet and dietary components may be beneficial not only on MS symptoms but also on disease progression as well as on disability status (8–10). The effect of single dietary components on MS pathogenesis has yet to be determined. Current hypotheses suggest an indirect role of dietary factors affecting cardiovascular risk (11), obesity (12), or lipid profile alterations (13). Interestingly, emerging evidence suggests that diet may play a role in determining (or preventing) a chronic immune system activation and inflammatory state (14). In this view, the role of diet in persons with MS (pwMS) may potentially play a more important role than originally thought, while understanding the mechanisms underlying such pro/anti-inflammatory activities might be useful to modulate and adjust eating habits and eventually modify the disease course.

Among the most recently studied factors potentially affecting the risk of degenerative diseases, the level of food processing gained particular attention (15). The NOVA classification (name is not an acronym) (16) groups foods, according to the nature, extent, and purpose of the industrial processing they undergo, into four classes: unprocessed or minimally processed foods, processed culinary ingredients, processed foods, and ultra-processed foods. Unprocessed (or natural) foods are edible parts of plants, animals, fungi, algae, and water, after separation from nature. Minimally processed foods are natural foods altered by processes like removal of inedible or unwanted parts, and drying, pasteurization, boiling, and so on, necessary to preserve them. Processed culinary ingredients (i.e. oils, butter, sugar, and salt) are substances derived from Group 1 foods or from nature by processes that include pressing, refining, grinding, milling, and drying. They are not meant to be consumed by themselves, and are normally used in combination with Group 1 foods to make freshly prepared drinks, dishes and meals. Processed foods, such as bottled vegetables, canned fish, fruits in syrup, cheeses, and freshly made breads, are made essentially by adding salt, oil, sugar, or other substances from Group 2 to Group 1 foods. Ultra-processed foods (UPFs) are “formulations of ingredients, mostly of exclusive industrial use, that result from a series of industrial processes (hence “ultra-processed”), many requiring sophisticated equipment and technology.” This group includes, for example, fast foods, ultra-processed dairy, breakfast cereals, biscuits, pastries, and cakes. UPFs are generally quite elaborated compared to unprocessed or minimally processed foods; they often are composed by ingredients not commonly used for home-made preparations (i.e., fructose-glucose syrup, added-sugar, maltodextrin, palm oil, hydrogenated oil, and food additives like artificial sweeteners, flavor enhancers) that give higher palatability to the product. In recent years, UPFs consumption increased worldwide (17), also due to their elevated market availability. Their massive introduction into popular diets has been hypothesized to potentially play a role in the increased prevalence of chronic non-communicable diseases, CVD, cerebrovascular disease, depression, obesity, and all-cause mortality in many countries (18, 19).

UPFs have been generally considered unhealthy due to their poor nutritional value: in fact, data from nationally representative samples revealed that these foods are typically high in calories, sugar, unhealthy fats and salt, and low in dietary fiber, vitamins, and minerals (20). However, emerging evidence suggests that factors other than their nutritional content (i.e., chemical additives) may play a role in human health, putting under the spot also those foods nutritionally adequate but yet ultra-processed, which may play an additional role toward chronic inflammation (21). Thus, the aim of this study was to investigate the potential association of UPF consumption and MS severity in a group of MS Italian consecutive patients.

### 1.1. Study design

This is a single-center, observational cross-sectional study conducted by Human Nutrition and Eating Disorders Research Center—University of Pavia in collaboration with C. Mondino National Neurological Institute, Pavia, Italy. The study was conducted according to the Declaration of Helsinki and was approved by the San Matteo Ethical Committee (P-20200064205, date: 08/05/2020).

## 2. Material and methods

### 2.1. Study population

Participants were recruited between September 2020 and March 2022 during their routine neurological control visits at Mondino Neurological Institute. The study objectives and procedures were explained to each participant and his/her written informed consent was obtained. Inclusion criteria were age > 18 years, MS diagnosis and ability to give verbal and written consent. Significant cognitive-cooperative impairment, lack of compliance, and presentation with primary progressive MS were considered exclusion criteria.

### 2.2. Data collection

Demographic (age, sex, marital status, educational level), neurological and nutritional (anthropometric measures, dietary habits) information were collected for each participant. Lifestyle characteristics (physical activity, smoking habits) were also investigated.

Marital status was classified in: (i) unmarried and widowed, (ii) married; educational level was categorized as: (i) low (primary/secondary), (ii) medium (high school), and (iii) high (university). Physical activity level was assessed through the International Physical Activity Questionnaire (IPAQ), which include a panel of questionnaires (five domains) investigating the time spent being physically active in the last week that allow categorizing participants in (i) low (ii) medium, and (iii) high physically active (22). Smoking status was classified as: (i) never smoke, (ii) current smoker, and (iii) former smokers.

Anthropometric measures were auto reported by the patients during a telephonic interview. It was not possible to visit patients in our Human Nutrition Center because enrollment started during the COVID-19 pandemic. Body Mass Index (BMI) was calculated as weight (kg): height (m^2^) and then patients were classified in normal-weight, overweight, and obese (no under-weight patients occurred in the study sample).

### 2.3. Dietary assessment

Food consumption was assessed through a validated Food Frequency Questionnaire (FFQ) consisting of 110 items (23), representative of the diet during the last 6 months. It was administered by an expert dietitian during a telephonic interview that took about 30–40 min. The FFQ investigated the consumption of 110 food and beverages groups for which the patients could choose between nine frequency options (“never,” “once a month,” “twice a month,” “once a week,” “2–3 times/week,” “4–5 times/week,” “once a day,” “2–3 times/day,” “4–5 times/day”). For each food item of the FFQ, the dietitian indicated the medium serving size; if the portion commonly consumed by the participant was not correspondent, frequency of consumption was modified accordingly. The average food consumption was calculated (in g or ml) by following the standard portion sizes and then converted in 24-h intake. Energy, macro and micro-nutrient intakes were obtained using standard food composition tables of the Italian Research Center for Foods and Nutrition^1^. The food items were grouped into groups according to the NOVA classification as follow: group 1, unprocessed or minimally processed foods (i.e., rice and other cereals, meat, fish, milk, eggs, fruit, vegetables, nuts, etc.); group 2, processed culinary ingredients (i.e., sugar, vegetable oils, and butter); group 3, processed foods (i.e., processed breads and cheese); group 4, UPFs (i.e., confectioneries, salty snacks, fast-foods, soft drinks, etc.). For the purpose of this study, the mean share of the NOVA group 1 (unprocessed/minimally processed foods) and NOVA group 4 (UPFs) to the total daily energy intake was estimated and participants dichotomized with the median energy shares of unprocessed/minimally processed and UPF intake as cut off to identify the variables of exposure.

### 2.4. Neurological assessment

The neurological disability of MS patients was quantified by the Expanded Disability Status Scale (EDSS), which rates seven neurological domains [Visual, Brainstem, Pyramidal (motor), Cerebellar (coordination), Sensory, Cerebral, and Bowel/bladder] in the context of a standard neurological examination. Ambulation scoring concludes evaluation. The final EDSS score is assigned according to the scores attributed to the single neurological systems.

The clinical impact of MS was calculated applying the Multiple Sclerosis Severity Score (MSSS) (24). MSSS represents the severity of MS at a given time; it is calculated using an algorithm that adjusts EDSS according to the corresponding disease duration.

We used Herbert's severity grading (25), based on the different values of the MSSS, to classify patients according to their disability status. Herbert divided MS patients into six approximately equipopulated groups of disability. Patients with an MSSS < 1.7 would be classified as having mild MS; patients with an MSSS between 1.7 and 3.4, moderate MS; patients with an MSSS from 3.4 to 5.0, intermediate MS; MSSS ranging between 5.0 and 6.7, accelerated MS; MSSS of 6.7 to < 8.3, advanced MS; and MSSS > 8.3, aggressive MS.

Based on the distribution of disease severity in the study sample, participants were categorized as “mild” (mean MSSS values < 1.7; *n* = 50), and “moderate to high” MS (mean MSSS values >1.7; *n* = 56).

### 2.5. Statistical analysis

Categorical variables are presented as frequencies of occurrence and percentages, with Chi-squared test used to assess differences between level of consumption of unprocessed/minimally processed and UPFs (low vs. high). Continuous variables are expressed as mean and standard deviations (SDs), with Student's *t*-test used to explore differences between groups of disease severity. The association between consumption of food groups by level of processing and SM severity was assessed by performing logistic regression analyses and calculation of odds ratios (ORs) and 95% confidence intervals (CIs) for an unadjusted model and a multivariate model adjusted for energy intake (continuous, kcal/d), age (continuous, years), sex (male, female), BMI (normal, overweight, obese), marital status (unmarried/widowed, married), educational status (low, medium, high), smoking status (never, current, former), and physical activity level (low, medium, high). All reported *P*-values were based on two-sided tests and compared to a significance level of 5%. SPSS 21 (SPSS Inc., Chicago, IL, USA) software was used for all the statistical calculations.

## 3. Results

A total of 130 participants were enrolled in the study. Five of them decided not to complete the telephonic interview, 15 had missing data, and four had primary progressive MS. The final sample was composed of 106 pwMS.

Baseline characteristics of the study sample on the basis of food consumption by degree of processing are described in Table 1. No statistical differences were found in food consumption according to demographics (age, sex, marital status, and educational level), BMI, physical activity and smoking status, neither for unprocessed/minimally processed, and UPFs.

**Table 1:** Baseline characteristics of the study sample according to consumption of foods by level of processing according to NOVA classification in the study sample (*n* = 106).

	**Unprocessed/minimally processed food consumption**		***P*-value**	**UPF consumption**		***P*-value**
	**Low**	**High**		**Low**	**High**	
**Age, mean (SD)**	49.5 (12.7)	49.7 (11.3)	0.936	49.8 (12.4)	49.4 (11.6)	0.888
**Sex**, ***n*** **(%)**			0.224			0.435
Men	22 (44.5)	16 (30.2)		16 (39.0)	22 (39.3)	
Women	31 (58.5)	37 (69.8)		34 (68.0)	34 (60.7)	
**Marital status**, ***n*** **(%)**			1.000			0.471
Unmarried/widowed	21 (39.6)	21 (39.6)		18 (36.0)	24 (42.9)	
Married	32 (60.4)	32 (60.4)		32 (64.0)	32 (57.1)	
**Educational level**, ***n*** **(%)**			0.734			0.303
Low	9 (17.0)	10 (18.9)		7 (14.0)	12 (21.4)	
Medium	28 (52.8)	24 (45.3)		23 (46.0)	29 (51.8)	
High	16 (30.2)	19 (35.8)		20 (40.0)	15 (26.8)	
**BMI**, ***n*** **(%)**			0.841			0.815
Normal-weight	36 (67.9)	35 (66)		35 (70.0)	36 (64.3)	
Overweight	14 (26.4)	16 (30.2)		13 (26.0)	17 (30.4)	
Obese	3 (5.7)	2 (3.8)		2 (4.0)	3 (5.4)	
**Physical activity level**, ***n*** **(%)**			0.499			0.109
Low	24 (45.3)	21 (39.6)		17 (34)	28 (50.0)	
Medium	20 (37.7)	18 (34.0)		23 (46)	15 (26.8)	
High	9 (17)	14 (26.4)		10 (20)	13 (23.2)	
**Smoking status**, ***n*** **(%)**			0.155			0.301
Never	26 (49.1)	34 (64.2)		31 (62.0)	29 (51.8)	
Current	11 (20.8)	11 (20.8)		11 (22.0)	11 (19.6)	
Former	16 (30.2)	8 (15.1)		8 (16.0)	16 (28.6)	

Table 2 shows the distribution of clinical parameters by consumption of food groups by degree of processing. There were no significant differences in terms of disease severity and course, current therapy, and type (first line vs. high potency).

**Table 2:** Clinical characteristics of the study sample according to consumption of foods by level of processing according to NOVA classification in the study sample (*n* = 106).

	**Unprocessed/minimally processed food consumption**		***P*-value**	**UPF consumption**		***P*-value**
	**Low**	**High**		**Low**	**High**	
**MSSS, mean (SD)**	2.85 (2.34)	2.89 (2.41)	0.923	2.62 (2.36)	3.09 (2.36)	0.308
**Disease course**, ***n*** **(%)**			0.814			0.383
Relapsing-remitting	41 (77.4)	42 (79.2)		41 (82.0)	42 (75.0)	
Secondary progressive	12 (22.6)	11 (20.8)		9 (18.0)	14 (25.0)	
**Current therapy**, ***n*** **(%)**			0.677			0.688
No	18 (34.0)	16 (30.2)		17 (34.0)	17 (30.4)	
Yes	35 (66.0)	37 (69.8)		33 (66.0)	39 (69.6)	
**Therapy type**, ***n*** **(%)**			0.190			0.123
First line	32 (84.2)	31 (72.1)		34 (85.0)	29 (70.7)	
High potency	6 (15.8)	12 (27.9)		6 (15.0)	12 (20.3)	

Tables 3, 4 show the consumption of major food groups and micro- and macronutrients in the whole sample and stratified by groups of MS severity. No substantial differences across individual food groups nor for nutrients were found.

**Table 3:** Mean daily intake (and standard deviations) of major food groups according to NOVA classification by multiple sclerosis status.

**NOVA food groups**	**Total**	**Multiple sclerosis severity**		***P*-value**
		**Moderate to high** **(*****n*** = **56)**	**Mild** **(*****n*** = **50)**	
	***Mean (SD), g/d***			
**Unprocessed or minimally processed foods**				
Red meat and poultry	48.3 (28.8)	49.2 (29.0)	47.1 (28.7)	0.719
Fish and sea foods	47.2 (35.0)	45.0 (33.1)	50.0 (37.5)	0.465
Milk and unprocessed dairy	104.6 (132.1)	118.2 (146.8)	86.9 (109.1)	0.227
Eggs	14.6 (20.2)	14.3 (18.6)	14.9 (22.3)	0.880
Grains and pasta	67.2 (39.2)	69.0 (42.4)	64.8 (34.9)	0.589
Fruits	391.0 (245.6)	373.4 (234.5)	413.8 (260.3)	0.404
Vegetables	217.1 (147.0)	198 (145.7)	242.0 (146.5)	0.127
Legumes	39.2 (44.7)	32.9 (43.7)	39.3 (46.4)	0.991
Salty snacks	3.4 (6.4)	3.8 (7.7)	2.9 (4.2)	0.493
Carbonated soft-drinks	23.7 (50.1)	20.3 (44.5)	28.3 (56.8)	0.416
Margarine	0.2 (1.1)	0.3 (1.4)	0.1 (0.2)	0.280
Confectioned juices	18.5 (50.3)	24.9 (59.5)	10.0 (33.7)	0.132
**Processed culinary ingredients**				
Vegetable oils	8.5 (2.8)	8.3 (3.0)	8.9 (2.6)	0.276
Animal fats	1.1 (2.3)	1.3 (2.5)	0.9 (2.0)	0.376
Table sugar	3.0 (3.7)	2.9 (3.5)	3.3 (3.9)	0.594
Fruit juice (natural)	39.5 (67.9)	42.6 (66.4)	35.3 (70.3)	0.588
**Processed foods**				
Breads	124.2 (99.7)	115.6 (106.4)	135.4 (90.1)	0.314
Cheese	33.5 (40.4)	37.1 (49.5)	28.7 (23.4)	0.286
Beer, wine, and liquors	64.0 (93.4)	56.3 (93.6)	74.1 (93.1)	0.333
Processed meats (cured)	11.6 (12.2)	12.1 (12.9)	11.0 (11.3)	0.634
**Ultra-processed foods**				
Fast foods	3.9 (8.9)	3.7 (8.7)	4.1 (9.3)	0.803
Ultra-processed dairy	42.1 (51.7)	40.3 (55.5)	44.4 (46.7)	0.683
Breakfast cereals	2.9 (7.2)	3.3 (8.1)	2.5 (6.0)	0.556
Biscuits, pastries, cakes	51.4 (41.4)	50.3 (42.8)	52.9 (39.8)	0.749
Confectionery and creams	0.4 (1.1)	0.5 (1.4)	0.3 (0.7)	0.492
Ice creams	31.9 (52.5)	39.5 (63.7)	22.0 (30.3)	0.089

**Table 4:** Mean daily intake (and standard deviations) of major macro- and micronutrients by multiple sclerosis status.

**Nutrients**	**Total**	**Multiple sclerosis severity**		***P*-value**
		**Moderate to high** **(*****n*** = **56)**	**Mild** **(*****n*** = **50)**	
	**Mean (SD)**			
Energy (kcal)	1,934.6 (604.7)	1,952.4 (668.3)	1,911.2 (516.3)	0.730
Proteins (g/day)	77.8 (26.8)	78.7 (29.4)	76.5 (23.4)	0.682
Lipids (g/day)	63.3 (24.8)	65.8 (27.8)	60.1 (20.0)	0.239
Cholesterol (mg/day)	162.2 (83.6)	171.2 (98.1)	150.4 (58.8)	0.205
Saturated fatty acids (%)	24.7 (12.1)	26.0 (14.0)	22.9 (9.0)	0.191
MUFA (%)	26.4 (9.3)	27.1 (10.5)	25.5 (7.5)	0.394
PUFA (%)	11.1 (4.9)	11.3 (5.5)	10.9 (4.2)	0.720
Carbohydrates (g/day)	274.2 (94.9)	272.2 (102.8)	276.9 (84.5)	0.801
Total fiber (g/day)	29.5 (13.5)	29.1 (14.5)	30.1 (12.2)	0.698
Sodium (mg/day)	2,181.7 (780.4)	2,128.3 (823.1)	2,251.3 (723.8)	0.424
Potassium (mg/day)	3,482.7 (1,300.6)	3,468.2 (1,387.6)	3,501.5 (1,192.4)	0.897
Iron (mg/day)	14.2 (5.6)	14.2 (6.2)	14.2 (4.8)	0.990
Calcium (mg/day)	826.8 (413.2)	868.4 (475.5)	772.5 (310.9)	0.238
Phosphorus (mg/day)	1,325.3 (491.8)	1,345.4 (543.0)	1,299.1 (420.2)	0.633
Magnesium (mg/day)	394.0 (147.4)	393.1 (158.0)	395.2 (134.2)	0.943
Zinc (mg/day)	10.9 (4.0)	11.1 (4.5)	10.7 (3.4)	0.734
Copper (mg/day)	2.0 (0.8)	2.0 (0.9)	2.0 (0.7)	0.929
Selenium (μg/day)	96.2 (39.2)	94.4 (42.7)	98.5 (34.6)	0.598
Thiamin (mg/day)	1.7 (0.8)	1.8 (1.0)	1.6 (0.6)	0.216
Riboflavin (mg/day)	2.1 (0.9)	2.2 (1.1)	2.1 (0.8)	0.358
Niacin (mg/day)	20.8 (7.1)	20.4 (7.3)	21.3 (6.8)	0.542
Pyridoxine—vitamin B6 (mg/day)	1,396.8 (423.8)	1,329.4 (413.0)	1,484.7 (426.1)	0.061
Vitamin A retinol eq. (mg/day)	879.5 (413.8)	865.6 (454.6)	897.6 (357.8)	0.696
Vitamin C (mg/day)	166.5 (92.4)	161.9 (83.5)	172.6 (103.6)	0.559
Vitamin E (mg/day)	8.7 (3.5)	8.7 (3.6)	8.7 (3.3)	0.947
Vitamin D (μg/day)	4.1 (3.1)	3.8 (2.7)	4.4 (3.6)	0.382
Vitamin B12 (μg/day)	5.1 (2.7)	5.4 (3.1)	4.6 (2.0)	0.122
Folate—vitamin B9 (μg/day)	372.8 (162.1)	363.9 (171.1)	384.6 (150.6)	0.517

The association between food consumption by degree of processing and MS severity is shown in Table 5. No association was found in the unadjusted and multivariate-adjusted regression analyses for unprocessed/minimally processed foods and MS severity. In contrast, higher UPF consumption was associated with moderate-to-high MS severity compared to lower consumption in both the unadjusted model (OR = 2.28, 95% CI: 1.04–5.01) and after adjustment for potential background (OR = 2.46, 95% CI: 1.04–5.83) and clinical confounding factors (OR = 2.97, 95% CI: 1.13–7.77).

**Table 5:** Association between food intake based on level of processing and moderate-to-high multiple sclerosis (as compared to mild).

	**Moderate-to-high multiple sclerosis, OR (95% CI)**	
	**Low consumption**	**High consumption**
**Unprocessed/minimally foods**		
Unadjusted	1	0.85 (0.39–1.85)
Multivariate^*^	1	0.98 (0.40–2.26)
Multivariate model 2^**^		0.97 (0.39–2.44)
**UPFs**		
Unadjusted	1	2.28 (1.04–5.01)
Multivariate model 1^*^	1	2.46 (1.04–5.83)
Multivariate model 2^**^	1	2.97 (1.13–7.77)

* Multivariate model 1 was adjusted for energy intake (continuous, kcal/d), age (continuous, years), sex (male, female), BMI (normal, overweight, obese), marital status (unmarried/widowed, married), educational status (low, medium, high), smoking status (never, current, former), and physical activity level (low, medium, high).

** Multivariate model 2 was further adjusted for disease course and therapy.

## 4. Discussion

In this study we investigated the relationship between UPFs consumption in a sample of consecutive pwMS and their disease severity, showing an association between higher intake of UPFs with worse disease severity. To our knowledge, this is the first study reporting such findings, representing an important hypothesis to be further investigated.

The associations between patterns of food intake and health in pwMS are of increasing global interest. Many studies explored the effects of nutrients and dietary patterns in populations with MS; however, the possible role of diet in disease progression is not well-understood (9). Previous studies investigating dietary habits in pwMS highlighted a tendency for high-sugars, high-fat and high-protein diets (26). Fitzgerald et al. (27) reported that pwMS with no history of previous dietary restrictions reported lower overall fruit and vegetable intake and higher consumption of red and processed meat, added-sugar, and sugar-sweetened beverages compared to individuals with a history of diet, resulting in a worse diet quality in pwMS. Another recent cross-sectional study of 261 pwMS found no association between dietary patterns and disability, quantified by the Extended Disability Status Scale (EDSS) (28). Both studies show some similarities with ours: (i) dietary habits were investigated through a Food Frequency Questionnaire, (ii) the samples were composed mainly by individuals with mild-to-moderate disease severity and relapsing remitting clinical course. There are also some differences, for example neurological disability was assessed through EDSS or PDDS (while we used MSSS as a comparator). Different results were reported by Silveira et al. (29). They examined the overall diet quality, through the Healthy Eating Index (HEI)-2015 score, in 128 wheelchair-bound patients with progressive MS. Dietary habits were investigated using both a food Frequency questionnaire (Diet History Questionnaire, DHQ-III) and a 24-h recall questionnaire (Automated Self-Administered 24-h, ASA24), whose results were used to calculate the HEI-2015 score. They found that pwMS patients had a better diet quality compared with the general american population, with adequate or moderate intakes of fruits, vegetables, greens and beans, dairy, total protein foods, sodium, added sugar, and saturated fats. No references were done to the adequacy of their intakes to the US recommendation. Another study (30), reported no remarkable differences in dietary habits between 34 healthy control subjects and 66 pwMS, except for higher consumption of carbohydrates in patients. When compared to the World Health Organization's recommended daily amounts, both groups had higher protein and lower carbohydrate intakes. Moreover, dietary habits of pwMS were not associated with their neurological disability, quantified by EDSS. In general, comparing results on dietary consumption in pwMS is not simple, because studies often use different approaches (i.e., dietary quality index, foods, nutrients) to evaluate both nutritional and neurological aspects, and often focus the attention on the possible differences between pwMS with different severity degree and healthy controls.

The results obtained in the current study suggest a possible impact of ultra processed foods on MS severity. It is not known how UPFs could determine this effect. Recents reviews (31, 32) introduced the mechanisms through which UPFs (and their constituents) may influence oxidative stress and the inflammatory response. Both these conditions could play an important role in modulating processes of the immune system (33), that is known to be involved in MS. UPFs are generally rich in saturated fats, free sugars, and sodium, while the intake of fiber, protein, potassium, and antioxidants are low. They also often contain high amounts of additives relative to minimally processed foods and the processings they undergo can determine the production of dangerous substances such as acrylamide, acrolein, polycyclic aromatic hydrocarbons, and furan. These products are related to alterations in the oxidative-reductive processes, leading to oxidative stress and the development of chronic diseases (34, 35). Juul et al. (36) reported that high and prolonged consumption of certain characteristic ingredients of an ultra-processed diet is a factor in the development of oxide-reductive alterations at the cellular level. A recent study on a wide Italian population (37) found that processed foods and UPFs were positively associated with a low-grade inflammation status. In addition, at a molecular level, some nutrient molecules upon binding to their target are able to activate signal transduction mechanisms that are involved in the immune system and inflammation (38). Moreover, nutrients also induce activation of specific transcription factors which are involved in gene expression of products implicated in the metabolism, either in the metabolism of the nutrient itself or in the energy homeostasis, with the resultant activation of anabolic or catabolic pathways (39). Saturated and trans-fatty acids, high content of glucose, salt, and alcohol have an activating effect on the NFkB, leading to a shift of the metabolic balance toward anabolism, associated with inflammation (39). All these factors may be a possible and partial explanation for our findings, but certainly further investigations are needed.

The present study has some limitations that need to be considered before drafting conclusions. First, the observational nature of the study does not allow to assess cause-effect relationships but only associations. Second, the recruiting strategy and the relatively small sample size does limit the possibility to generalize the results to all MS patients, especially if recruited in other geographical areas with different dietary habits. Third, the dietary habits have been assessed through FFQs, which may suffer from over- or under-reporting due to recall bias or social desirability. Fourth, the NOVA classification is by far the most used classification to identify the level of food processing; however, there are concerns regarding its actual ability to discern across food groups nor to best identify food groups that are effectively detrimental for human health. Fifth, although we investigated several background characteristics and assessed that they were not related to the variable of interest, we cannot rule out the possibility of other unexplored confounding factors. Furthermore, we did not investigate the presence or absence of comorbidities, specifically the cardiovascular ones, which are known to be related both to a worst MS outcome and to an increased UPFs consumption.

## 5. Conclusions

In conclusion, higher intake of UPFs was associated with greater odds of higher MS severity. Although these results are only preliminary and hypothesis generating, it is important to explore how various aspects of the diet may relate to MS severity in order to identify the best strategy to support MS patients over the disease course.

## Data availability statement

The datasets presented in this study can be found in online repositories. The names of the repository/repositories and accession number(s) can be found in the article/supplementary material.

## Ethics statement

The studies involving human participants were reviewed and approved by San Matteo Ethical Committee (P-20200064205, date: 08/05/2020). The patients/participants provided their written informed consent to participate in this study. Written informed consent was obtained from the individual(s) for the publication of any potentially identifiable images or data included in this article.

## Author contributions

MG and GG: conceptualization. MG, GG, and AT: methodology. MG, CF, ET, RB, and ALM: investigation. MG, ET, RB, ALM, and GG: data curation. MG: writing—original draft preparation. MG, GG, AT, ET, RB, ALM, A-QW, and CF: writing—review and editing. AT: supervision. All authors have read and agreed to the present version of the manuscript.

## Footnotes

^1^Available online at: https://www.alimentinutrizione.it/sezioni/tabelle-nutrizionali (accessed July 16, 2022).
